# Chronic Hyperhomocysteinemia Impairs CSD Propagation and Induces Cortical Damage in a Rat Model of Migraine with Aura

**DOI:** 10.3390/biom14111379

**Published:** 2024-10-29

**Authors:** Elena Gerasimova, Daniel Enikeev, Aleksey Yakovlev, Andrey Zakharov, Guzel Sitdikova

**Affiliations:** 1 Department of Neuroscience, Sirius University of Science and Technology, 354340 Sirius, Russia; danielveenik@gmail.com; 2 Institute of Translational Biomedicine, Saint-Petersburg State University, 199034 Saint-Petersburg, Russia; 3 Pavlov Institute of Physiology, Russian Academy of Sciences, 199034 Saint-Petersburg, Russia; 4 Department of Human and Animal Physiology, Institute of Fundamental Medicine and Biology, Kazan Federal University, 18 Kremlevskaya Str., 420008 Kazan, Russia; alv.yakovlev@gmail.com (A.Y.); sitdikovaguzel@gmail.com (G.S.); 5 Department of Normal Physiology, Kazan State Medical University, 49 Butlerova Str., 420012 Kazan, Russia; anvzaharov@kpfu.ru; 6 Department of Medical Physics, Institute of Physics, Kazan Federal University, 16a Kremlyovskaya Str., 420008 Kazan, Russia

**Keywords:** migraine with aura, hyperhomocysteinemia, cortical spreading depression, somatosensory cortex, lactate dehydrogenase, cortex viability

## Abstract

Hyperhomocysteinemia (hHCY) is a metabolic disorder characterized by elevated levels of homocysteine in plasma. hHCY correlates with a high risk of migraine headaches, especially migraine with aura. Cortical spreading depression (CSD) is a wave of depolarization passing through neurons and glial cells of the cortex and is considered an electrophysiological correlate of migraine aura. The aim of the present study was to analyze neuronal activity and CSD in the somatosensory cortex of rats in vivo with prenatal hHCY and to assess cortex viability after 2 h of CSD generation. Female rats were fed a diet high in methionine, and their offspring with high homocysteine levels in plasma were further used in experiments. Recurrent CSD was evoked by local KCl application on the dura surface. Neuronal viability was assessed by measuring the activity of lactate dehydrogenase (LDH) in the brain and 2,3,5-triphenyltetrazolium chloride staining of the somatosensory cortex after two hours of CSD generation. Animals with hHCY exhibited higher neuronal activity, and more CSDs were generated in response to KCl, indicating higher cortical excitability. Propagation of recurrent CSD was impaired in supragranular cortical layers, and the recovery of multiple unit activity and evoked sensory potentials after CSD was delayed in the hHCY group. Finally, in animals with prenatal hHCY, an ischemic focus was identified as a consequence of multiple CSDs, along with elevated levels of LDH activity in brain tissues, suggestive of diminished neuronal viability. These findings imply that prolonged elevated levels of homocysteine may not only predispose to migraine with aura but also potentially elevate the risk of migrainous infarction.

## 1. Introduction

Homocysteine is a sulfur-containing amino acid, an intermediate of methionine catabolism, and a substrate for the production of cysteine, pyruvate, and hydrogen sulfide (H_2_S) [1,2]. Homocysteine can be metabolized through the transsulfuration pathway or remethylated back to methionine via the remethylation pathway. In the transsulfuration pathway, homocysteine forms cystathionine via cystathionine β-synthase (CBS), with pyridoxal phosphate (PLP) acting as a cofactor. Alternatively, homocysteine can be remethylated to methionine through a folate-dependent reaction requiring the enzymatic activities of methionine synthase (MS) and 5,10-methylenetetrahydrofolate reductase (MTHFR) [3]. A number of factors, ranging from genetic mutations to impaired metabolism of folic acid and methionine and deficiency of vitamins B12 and B6, lead to an increase in homocysteine levels in plasma called hyperhomocysteinemia (hHCY) [4,5,6,7,8,9]. Plasma homocysteine levels ranging from 15 to 30 µM cause mild hHCY, 31 to 100 µM—moderate hHCY, and >100 µM—severe hHCY symptoms [10].

Several strategies have been applied in order to induce hHCY in animals, including dietary manipulation like excess consumption of L-methionine, diets deficient in B-vitamins, injection of homocysteine, or genetic models based on a mutation in CBS or MTHFR [11]. In addition, hHCY may be primarily induced in dams with further analysis of their offspring [11]. In this model, rats with maternal or prenatal hHCY demonstrated developmental impairments in early postnatal life, poor cognitive performance, and motor dysfunctions in postnatal ontogenesis. Importantly, rats with prenatal hHCY displayed an elevated homocysteine level throughout life, even without additional methionine supplementation due to an impaired methionine/homocysteine metabolic cycle in the perinatal period [12].

One of the most common neurological diseases associated with hHCY is migraine, characterized by higher neuronal excitability and neurovascular pathologies [13]. About 30% of migraineurs experience transient neurological deficits, including sensory (mainly visual) and motor disturbances, which may precede or overlap the headache phase [14]. Clinical studies demonstrated a positive relationship between levels of homocysteine in plasma or cerebrospinal fluid and frequency or headache severity of migraine attacks, especially migraine with aura [15], while the presence or absence of aura was independent of homocysteine levels in women [16]. Experimental studies indicated that rats with prenatal hHCY showed mechanical allodynia, photophobia, and anxiety and were more susceptible to the nitroglycerine administration chronic model of migraine [17,18].

The main physiological mechanism underlying aura is suggested to be cortical spreading depression (CSD) [19]. CSD is a wave of massive depolarization passing through neurons and glial cells of the cortex, causing significant changes in the transmembrane ion gradient [20] and followed by the long-term suppression of neuronal activity. This phenomenon is closely associated with many neurological disorders, including migraines, concussions, stroke, and subarachnoid hemorrhage [21].

Migraine, especially migraine with aura, is a risk factor associated with cerebral ischemia and ischemic stroke [22]. Migrainous infarction is a rare type of ischemic stroke and accounts for about 0.8 percent of all strokes. According to the International Classification of Headache Disorders, at least a 60 min aura attack and a premorbid migraine with aura diagnosis are necessary for the diagnosis of migrainous infarction [23,24]. Factors that contribute to migrainous infarction also include genetic and hormonal fluctuation, endothelial dysfunctions, and the use of antimigraine drugs, such as ergot alkaloids and triptans, causing vasoconstriction [25]. hHCY is suggested to be a comorbid factor of ischemic damage in animal models of human stroke [26,27] and at the same time increases the sensitivity of the somatosensory cortex to CSD [17]. However, the functional changes in cortical neurons of rats with hHCY during recurrent CSD generation were not investigated.

The aim of the present study was to analyze (1) the generation of CSD during 2 h of KCl application in the somatosensory cortex of rats with prenatal hHCY; (2) the neuronal activity and sensory evoked potentials before and after CSD; (3) the viability of the cortex after 2 h of CSD generation and the activity of lactate dehydrogenase (LDH) in brain tissues.

## 2. Materials and Methods

### 2.1. Animals

The studies were carried out on male Wistar rats (P45–90) (P0 = day of birth) in accordance with the EU Directive 2010/63/EU for animal experiments and the Local Ethics Committee of the Kazan Federal University (protocol No. 8 of 5 May 2015, No. 33 of 25 November 2021) and Sirius University of Science and Technology (protocol No. 2 of 2 May 2023). The animals were housed in polyethylene cages at room temperature (22 °C) with a 12 h light/dark cycle (lights on at 6 a.m.) and free access to food and water.

### 2.2. The Model of Prenatal Hyperhomocysteinemia

Control group rats (n = 12) were bred from females receiving a normal diet. Rats with prenatal hHCY were born from females fed a high-methionine diet (7.7 g/kg meal) three weeks before and during pregnancy and until the end of milk feeding (n = 10). The introduction of a male rat to a female obtaining a high-methionine diet was carried out only in the case of increased homocysteine levels in plasma after three weeks of methionine consumption. The plasma homocysteine concentration was measured by spectrophotometry using an ELISA reader (Multiskan FS, Thermo Fisher Scientific, Waltham, MA, USA) and the Homocysteine Colorimetric Assay Kit (E-BC-K143, ElabScience, Houston, TX, USA). The plasma homocysteine concentration in females on a methionine diet was elevated up to 34.5 ± 8.1 μM (n = 10), compared to control females—6.3 ± 1.0 μM (n = 12). In the offspring of the control group, the homocysteine level was 7.4 ± 1.2 μM (n = 12), and of the hHCY group—29.9 ± 4.5 μM (n = 13). Male rats were used in experiments to avoid the effects of hormonal changes due to the estrous cycle in females.

### 2.3. Surgery

**Figure 1:**
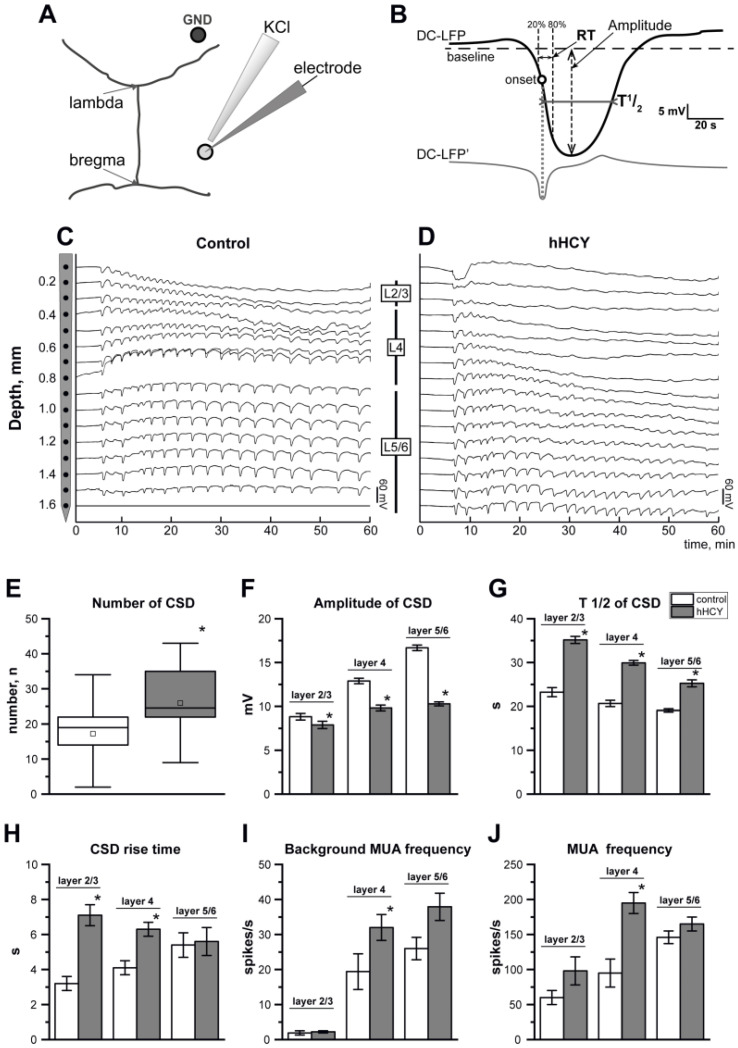
Cortical spreading depression (CSD) in the somatosensory cortex of rats. (**A**) Experimental design. CSD was recorded using a 16-site linear silicon electrode inserted in the somatosensory cortex; KCl (1 M) was applied into the same hole. The ground electrode (GND) was placed in the visual cortex. (**B**) Schematic trace of CSD (DC-LFP, top black trace) and its first derivative (DC-LFP’, bottom gray trace) with the following analyzed parameters: onset (the negative peak time of the first LFP derivative), amplitude (the maximal negative LFP peak from the baseline), half duration (T1/2, duration at half amplitude), rise time (RT, time between 20% and 80% of the CSD amplitude). The horizontal dashed line shows the baseline. (**C**,**D**) Original recordings of recurrent CSD using a 16-site linear silicon probe (the distance between recording sites is 100 μm) in all layers of the cortical column in control (**C**) and hHCY (**D**) rats. The number of CSDs generated during 2 h of KCl application (**E**), amplitude of CSD (**F**), duration at half amplitude of CSD (T1/2, (**G**)), CSD rise time (**H**) in the control (white columns) and hHCY (gray columns) groups. (**I**) Background MUA frequency in cortical layers 2/3, 4, and 5/6 in the control and hHCY groups. (**J**) The increment of MUA frequency in cortical layers 2/3, 4, and 5/6 at the onset (10 s before and 10 s after) of CSD compared to the background level in the control and hHCY groups. * *p* < 0.05 compared to the control group.

A standard protocol for preparing the rodents for surgery by inhalation (5% induction and 1.5–2% for maintenance) of the anesthetic isoflurane (Baxter, Deerfield, USA) was used, as described previously [17,18]. Under anesthesia, the skin and muscles were carefully cut and pulled apart on the surface of the skull. Then, anesthesia was switched to urethane using intraperitoneal injections (1.5 g/kg i.p.), and the animal head was fixed in the frame of a stereotaxic apparatus by the attached bars. On the left side of the skull, a 2 mm diameter hole was drilled above the somatosensory cortex (AP-2.0 to −2.5 mm; 5 to 6.5 mm lateral from the bregma). A ground electrode—a chlorine silver wire—was implanted in the cerebellum or visual cortex (Figure 1A). A self-regulating heating pad (TCAT-2LV controller, Physitemp Instruments LLC, Clifton, New Jersey, NJ, USA) was used to keep the body temperature at 37 ± 0.5 °C.

### 2.4. Electrophysiological Recordings

Extracellular local field potential (LFP) and multiple unit activity (MUA) were recorded by a 16-site linear silicon probe (Neuronexus Technologies, Ann Arbor, MI, USA), with 100 µm between recording sites, in direct current (DC) mode (input range = ±131 mV) using a DigitalLynx system (Neuralynx, Bozeman, MO, USA) and a bandpass range of 0–9000 Hz [17,28]. The record was digitized at 32 kHz and was analyzed in the MATLAB environment. The probe was inserted into the cortex at a depth of 1700 ± 100 µm. The cortex was given 40 min to recover before the recordings began. Stimulation of the whisker was carried out by a system where a needle (22 G) was attached to a piezoelectric bending actuator (Noliac, Kvistgård, Denmark) to which rectangular pulses of 200 ms duration were delivered at intervals of 5–10 s. The whiskers were trimmed to 1.0–1.5 mm, and the whisker was then placed into a blunt needle tip, which deflected it.

### 2.5. Data Analysis

CSD is consisted of depolarization waves propagated across the layers of the cortical column: supragranular (L2/3), granular (L4), and infragranular (L5/6). CSD detection was performed visually according to the specific LFP. For each channel, the baseline level was determined at the 20–10 s time window before the CSD. Preliminarily, CSD was detected by visual control, and the precise onset of CSD was calculated [28]. For each recording site, the local negative peak time of the first LFP derivative within the 20 s time window preceding the negative CSD peak was considered at the CSD onset. The amplitude–temporal parameters of the first CSD in each experiment were analyzed. The CSD amplitude as the maximal negative LFP peak from the baseline, CSD half duration (T1/2) as the CSD duration at its half amplitude, and rise time (RT), the time between 20% and 80% of the CSD amplitude, were calculated (Figure 1B).

In addition, the number and duration of CSD generation were determined. Local mechanical stimulation of the whisker evoked complex responses—sensory evoked potentials (SEPs) in the topographic locus of the rat somatosensory cortex. SEPs were identified as the first LFP troughs of sensory evoked responses. At the point where the SEP front and baseline collided, the SEP onset was found.

The peak of the SEP amplitude corresponding to the most negative LFP within 100 ms after the stimulus and the latency corresponding to the time from the stimulus to the SEP peak (SEP peak latency) were determined.

The principal vibrissa was determined as the whisker that produced the largest amplitude and shortest latency response. Recordings from the barrel cortex were made contralateral to vibrissae stimulation. CSD was initiated by the application of a KCl solution (10 µL) at a concentration of 1 M into the hole and was recorded for 2 h.

The current source density for SEPs was calculated as the second spatial derivative of the LFP for each recording site (upper and lower recording sites were excluded), using linear interpolation, and displayed as pseudocolor images [29]. The current source density was calculated after subtracting the baseline (a 1 s segment of the average LFP before the stimulus).

The wide-band signal was filtered (bandpass 300–3000 Hz), and negative events with amplitudes more than five standard deviations were considered as MUA. The frequency of MUA in cortical layers was calculated by averaging the frequency of MUA between channels located in the specific layer.

### 2.6. Histological Staining

2,3,5-Triphenyltetrazolium chloride (TTC) was used to assess and visualize neuronal damage in the brain after KCl-induced CSD for 2 h. After the experiment, animals were deeply anesthetized with urethane (3 g/kg, i.p.) and intracardially perfused with NaCl 0.9%. Then, rats were decapitated, and brains were extracted from the cranium.

Thalamocortical slices with a thickness of 400 μm were prepared on a vibratome, the Microm HM 650 V (Microm International GmbH, Walldorf, Germany), and then incubated in TTC (1%, phosphate-buffered saline solution, PBS) warmed up to 37 °C for 10 min, and washed in PBS afterward. Microphotographs of TTC-stained slices were obtained using an SZX16 wide zoom stereo microscope equipped with an SDF PLAPO 1 × PF objective and SZX2-ILLT LED transmitted light illumination base (Olympus, Tokyo, Japan). The images were acquired at 0.7×−1× magnification using an XC50 CCD camera (Olympus, Tokyo, Japan) at 2576 × 1932 pixel resolution. The volume of damaged tissue was calculated using the lesion area in successive slices estimated using FIJI ImageJ(NIH, Bethesda, MD, USA).

### 2.7. Brain Tissue Processing and Determination of Lactate Dehydrogenase (LDH) Activity

For biochemical studies, the brains of P30-P40 rats were used. Rats were sacrificed using mild isoflurane anesthesia followed by cervical dislocation. The cortex, hippocampus, and cerebellum of rats were quickly removed and dropped in ice-cold lysis Buffer (IS007, Cloud-Clone Corp., Wuhan, China) with a Dounce homogenizer containing a protease inhibitor cocktail tablet (S8820, Sigma-Aldrich, St. Louis, MO, USA). Tissue homogenates were centrifuged at 12,600 rpm for 15 min and stored at −20 °C.

LDH activity in serum was detected using a commercial enzyme kit (“LHD-2 Olveks”, Olveks-Diagnisticum, St Peterburg, Russia) by spectrophotometry at 340 nm using an ELISA reader (Multiskan FS microplate reader, Thermo Fisher Scientific, Waltham, MA, USA) in accordance with the manufacturer’s protocol. Lactate levels were expressed as arbitrary units per liter (au/L).

### 2.8. Statistical Analysis

The processing of experimental data was performed using specially developed software based on MATLAB (MathWorks, Natick, MA, USA)—ExpressAnalysis and Eview [30] (Andrey Zakharov, https://github.com/AndreyZakharovExp (accessed on 23 February 2024)) and OriginPro (OriginLab Corporation, Northampton, MA, USA). The Mann–Whitney test and Kruskal–Wallis test were used for the comparison of independent samples and the Wilcoxon test for related samples. Differences were considered statistically significant at *p* < 0.05. All results are presented as M ± m, where M is the mean, and m is the error of the mean.

## 3. Results

### 3.1. CSD and MUA in the Somatosensory Cortex of Rats with hHCY

The application of KCl on dura mater induced CSD across the layers of the somatosensory cortex (S1) recorded using a 16-site linear silicon probe (Figure 1C,D). The period of CSD generation in the control group was 86 ± 7 min (n = 13) and 69 ± 9 min in the hHCY group (n = 11, *p* > 0.05). At the same time, the number of CSDs generated during 2 h of KCl application was higher in the hHCY group (26 ± 30 versus 19 ± 2 in the control, *p* < 0.05) (Figure 1E).

Propagation of repetitive CSD across the layers of the somatosensory cortex was different in the control and hHCY groups during 2 h (Figure 1C,D). In the control group (n = 6), in four experiments, CSD was generated in the supragranular layers and spread to the infragranular layers for two hours; in two experiments, CSD propagation was limited by supragranular layers during the second hour of recordings (Figure 1C). In the hHCY group, CSD was initially generated in supragranular layers and propagated into the deeper layers (n = 10). However, in six rats, subsequent CSDs were limited by L4 after 15–20 min and by L5-6 after 30–40 min of recordings (Figure 1D).

The time–amplitude parameters of CSD were different in the two groups of animals. In the control, the amplitude of CSD was in L2/3—8.8 ± 0.3 mV, L4—12.9 ± 0.3 mV, and L5/6—16.7 ± 0.3 mV. In the hHCY group, the amplitude of CSD was lower in all cortical layers: in L2/3—7.8 ± 0.4 mV, L4—9.8 ± 0.3 mV, and in L5/6—10.3 ± 0.2 mV (*p* < 0.05) (Figure 1F). The duration of CSD (T1/2) calculated at half of the amplitude was significantly larger in the hHCY group. In the control, the duration of CSD was in L2/3—23.2 ± 1.03 s, in L4—25.6 ± 0.7 s, and in L5/6—19.01 ± 0.3 s. In the hHCY group, the duration of CSD was in L2/3—36.1 ± 1.7 s, in L4—38.4 ± 3.01 s, and in L5/6—36.9 ± 1.7 s (*p* < 0.05, Figure 1G). The rise time of CSD was in L2/3—3.2 ± 0.5 s, L4—4.1 ± 0.4 s, and in L5/6—5.4 ± 0.7 s. In the hHCY group, the rise time of CSD was in L2/3—7.1 ± 0.6 s (*p* < 0.05), L4—6.3 ± 0.4 s (*p* < 0.05), and L5/6—5.6 ± 0.8 s in (*p* > 0.05) (Figure 1H).

MUA as an index of neuronal excitability was assessed in L2/3, L4, and L5/6 of the barrel cortex in the control (n = 10) and hHCY groups (n = 11). The background neuronal activity was higher in the hHCY group—MUA frequency was in L2/3 2.2 ± 0.3 s^−1^ compared to the control—1.9 ± 0.6 s^−1^ (*p* > 0.05), in L4—32.0 ± 3.7 s^−1^ compared to the control—19.4 ± 5.1 s^−1^ (*p* < 0.05), and in L5/6—37.9 ± 3.9 s^−1^ compared to the control—26.0 ± 3.2 s^−1^ (*p* < 0.05) (Figure 1I). The onset of CSD was characterized by an initial increase in MUA, which was higher in L4 in the hHCY group. In the hHCY group, the increase in MUA in L2/3 was 98 ± 20 s^−1^ compared to 60 ± 10 s^−1^ in the control (*p* > 0.05), in L4 195 ± 15 s^−1^ compared to 95 ± 20 s^−1^ in the control (*p* < 0.05), and in L 5/6—165 ± 10 s^−1^ compared to 146 ± 9 s^−1^ in the control (*p* > 0.05) (Figure 1J).

### 3.2. Sensory Evoked Potentials in Rats with hHCY

**Figure 2:**
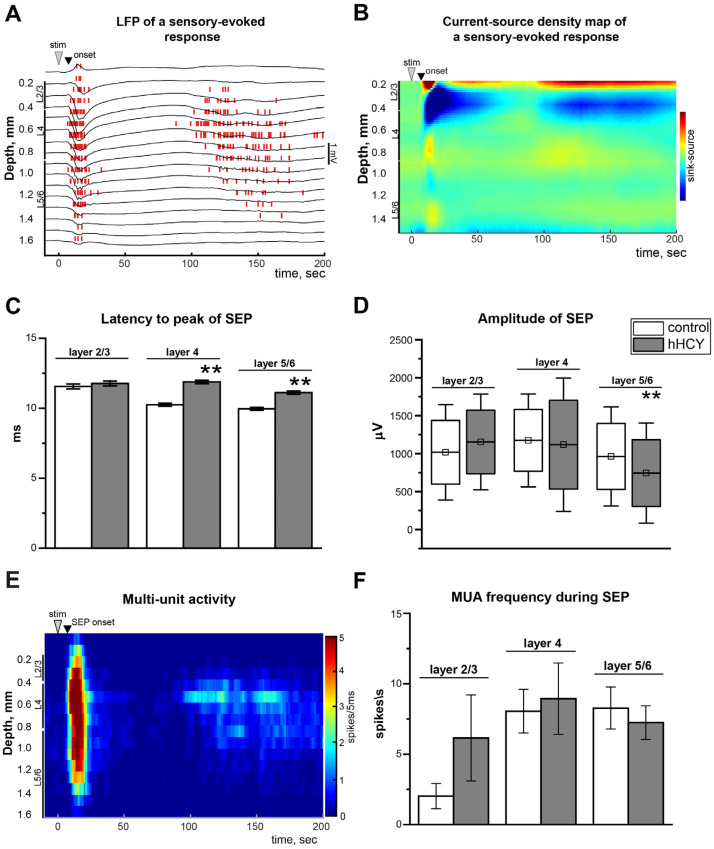
Sensory evoked potentials (SEPs) and MUA during and after SEPs in the rat cortex before CSD. (**A**) Individual example of SEPs in the rat barrel cortex caused by deflection of the principal whisker (stim). The MUAs marked by vertical red lines were detected as negative events exceeding eight standard deviations in amplitude. (**B**) Current source density map of an average SEP in the control. (**C**) Latency to peak of SEP (time from the onset to the maximal amplitude of SEP) in cortical layers 2/3, 4, and 5/6 in the control (white column) and hHCY (gray column) groups. (**D**) Amplitude of SEP in cortical layers 2/3, 4, and 5/6 in the control (white boxplot) and hHCY (gray boxplots) groups. On each box, the small square indicates the mean, horizontal lines indicate the median, the bottom and top edges of the box indicate the 25th and 75th percentiles, respectively, and the whiskers show the SD. (**E**) The panel depicts depth profiles for MUA during and after SEPs. Color-coded images represent the MUA frequency. The colors indicate the strength of the reaction, determined by the colored bars at the right of the panel. (**F**) MUA frequency in cortical layers 2/3, 4, and 5/6 in the control (white column) and hHCY (gray column) groups during SEPs. ** *p* < 0.01 compared to the control group.

SEPs were recorded in five control and seven hHCY rats. Short-term deflection of the principal vibrissa resulted in specific LFPs with a latency of 5 to 11 ms after stimulation, and the amplitude varied from 500 to 1300 µV in different cortical layers (Figure 2A). This depth profile picture of SEPs is typical for rats of the studied age and corresponds to the previous data [31]. The analysis of current source density demonstrated that the main sink of SEPs was L2/3, where most neurons were excited during sensory stimuli and did not differ between the experimental groups (Figure 2B).

The comparison of depth profiles of SEPs in the control and hHCY groups revealed the differences in the latency to peak and amplitude of SEPs (Figure 2C,D). Latency to peak in the control group in L2/3 was 11.55 ± 0.17 ms (control) and 11.76 ± 0.17 ms (hHCY, *p* > 0.05), in L4 it was 10.24 ± 0.10 ms (control) and 11.87 ± 0.12 ms (hHCY, *p* < 0.01), and in L5/6—9.95 ± 0.10 ms (control) and 11.11 ± 0.11 ms (hHCY, *p* < 0.01) (Figure 2C).

In L2/3, the SEP amplitude was 1017.39 ± 46.55 μV (control) and 1153.77 ± 24.28 μV (hHCY, *p* > 0.05), in L4 it was 1174.42 ± 23.58 μV (control) and 1117.42 ± 36.36 μV (hHCY, *p* > 0.05), and in L5/6—962.56 ± 24.59 μV (control) and 743.31 ± 24.59 μV (hHCY, *p* < 0.01) (Figure 2D). MUA was analyzed during SEPs (in the interval 0–20 ms from onset) and after SEPs (in the interval 20–520 ms from onset) and did not reveal differences between control and hHCY groups (Table 1, Figure 2E,F).

KCl application at the surface of dura mater, together with the generation of CSD, induced the suppression of SEPs in both groups of animals. After the first wave of CSD, the amplitude of SEPs in L4–L6 was restored during 3–5 min to the initial levels in four of five control rats (80% of animals), and in three of seven hHCY rats (43% of animals). MUA recovered up to 70–100% in five control animals; at the same time, in the hHCy group, MUA recovered only to 5–20% of the initial level in seven animals. Further, during repetitive CSD (from 50 to 100 min), SEPs were not recorded in four of five control rats and in five of seven hHCY rats. Similarly, MUA suppression was observed in all cortical layers.

**Figure 3:**
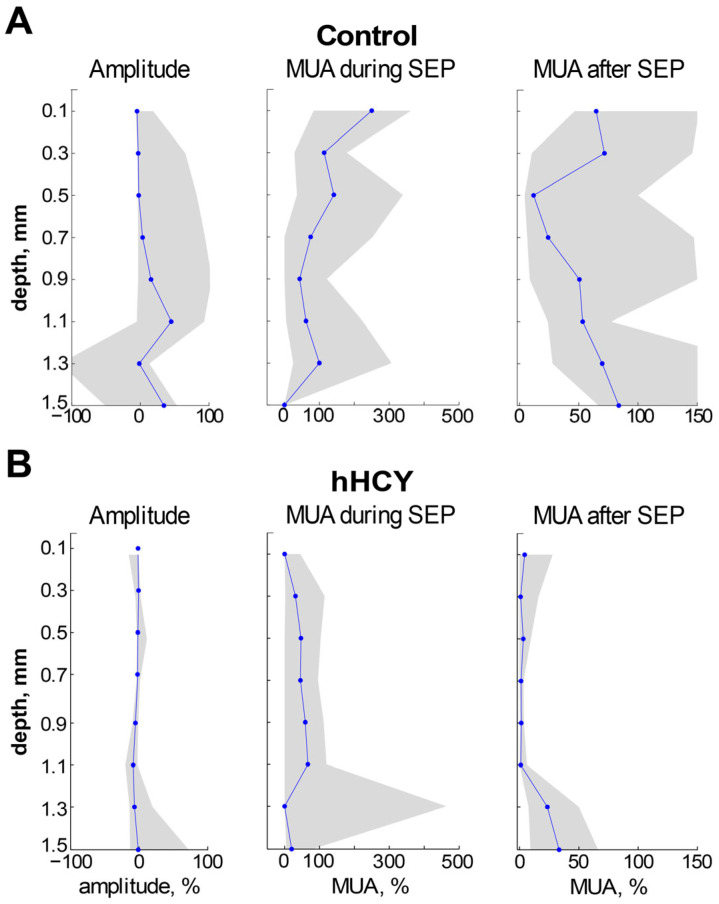
SEP and MUA depth profiles in min after repetitive CSD in the rat cerebral cortex. Recovery of parameters SEP and MUA after CSD, presented as the ratio of parameters before CSD to the value of parameters after CSD during the recovery period (5 min after the last CSD) in control (**A**) and hHCY (**B**) animals. The value of 100% was taken as the value of the parameters before generating the CSD. The blue line is the median and the gray zones are the boundaries of the interval from the 25th to the 75th percentile.

The depth profile recovery of the parameters of the MUA and SEP was assessed 5 min after the last CSD (Figure 3A,B). In the hHCY group, the recovery of SEP and MUA was almost negligible compared to the control.

**Figure 4:**
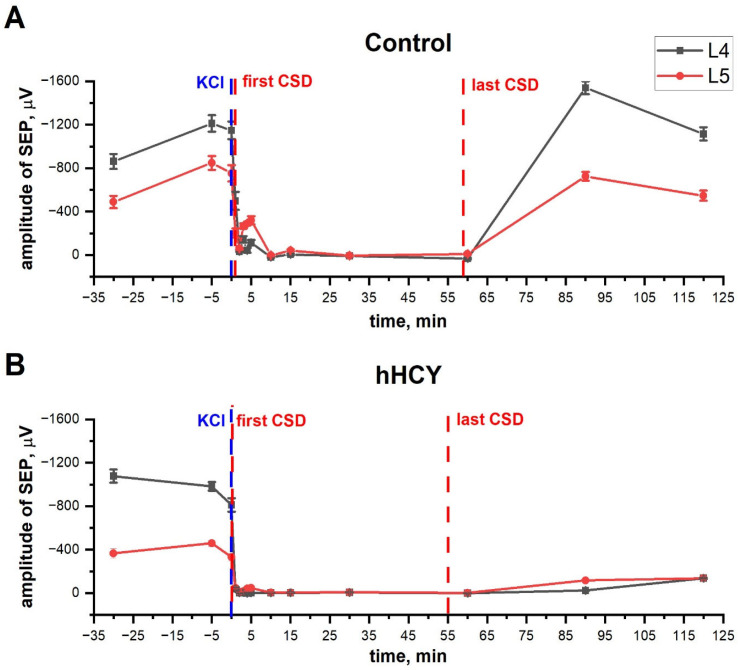
Recovery of SEP after CSD in control and hHCY rats. Individual examples of SEP amplitude during and after CSD in L4 and L5 layers of the cerebral cortex in control (**A**) and hHCY (**B**) animals. The time of KCl application is indicated by a blue dotted line, and the first and last CSD by a red dotted line.

Thirty minutes after the last CSD, the amplitude of SEPs achieved 90–110% of the initial levels in four control animals, whereas no recovery was found in the hHCY group (Figure 4). Similarly, MUA recovery (during and after SEPs) was observed in all experiments of the control group, while no recovery was found in the hHCY group.

### 3.3. TTC Staining of the Somatosensory Cortex After CSD Generation in Rat Brain

**Figure 5:**
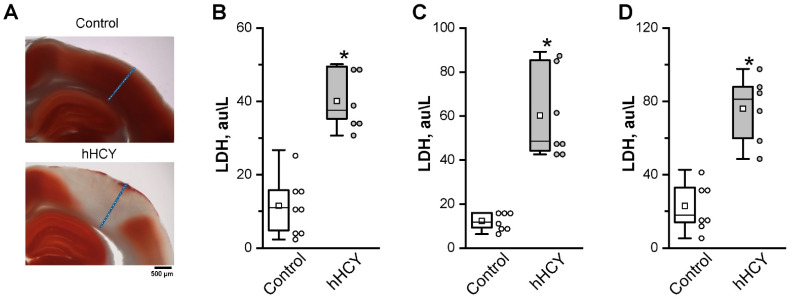
TTC staining of the somatosensory cortex and LDH activity in brain tissues. TTC-stained coronal sections of S1 of control and hHCY animals (**A**). The red portions show healthy tissue, and the white portion represents the necrotic infarction area. The electrode trace is depicted schematically at the site where the electrical activity of the brain was recorded. The activity of lactate dehydrogenase (LDH) in the homogenate of the rat cortex (**B**), cerebellum (**C**), and hippocampus (**D**) in rats of the control and with prenatal hHCY. One circle indicates one animal. * *p* < 0.05 compared to the control group.

To assess the viability of cortical neurons after 2 h of KCl exposure and multiple CSD generations, brain sections were stained with TTC dye. TTC is reduced by dehydrogenases in the mitochondria of living cells, turning them red; therefore, the activity of dehydrogenases is used to qualitatively assess neuronal viability [32]. In the somatosensory cortex of rats from the hHCY group, the non-colored area was observed in the site of CSD recordings—the so-called “necrotic funnel” or “ischemic focus”, with a volume of 68.4 ± 19.9 mm^3^ (in four of six animals). In the control group, the somatosensory cortex was stained evenly red (n = 5) (Figure 5A).

### 3.4. Lactate Dehydrogenase Activity

Neural cell injury was quantified by the measurement of LDH released from damaged cells into the extracellular fluid [33]. The results presented in Figure 5B–D indicated a significant increase (*p* < 0.05) in LDH activity in tissue homogenate of the rat brain from the hHCY group when compared to that of control rats.

In control conditions, the LDH activity in the cortex was 11.5 ± 2.8 au/L (n = 8), in the cerebellum—12.3 ± 1.5 au/L (n = 7), and in the hippocampus—23.1 ± 4.9 au/L (n = 7). In rats from the hHCY group, the LDH activity was higher in all samples: 40.1 ± 3.3 au/L in the cortex (*p* < 0.05, n = 6), 59.7 ± 8.8 au/L in the cerebellum (*p* < 0.05, n = 7), and 76.1 ± 7.6 au/L in the hippocampus (*p* < 0.05, n = 6).

## 4. Discussion

In this study, we provided a detailed analysis of brain electrical activity in multiple layers of the somatosensory cortex of rats with prenatal hHCY during recurrent CSD as a model of migraine with aura. The neuronal activity of rats with hHCY was observed to be higher, and a greater number of CSDs were generated in response to the application of KCl. This indicated that the cortical excitability of these rats was higher. At the same time, the propagation of recurrent CSD was impaired in supragranular cortical layers, and the recovery of MUA and evoked sensory potentials after CSD were negligible in the hHCY group. Finally, an ischemic focus was found in animals with hHCY as a result of multiple CSDs, which, together with increased levels of LDH activity in brain tissues, indicated reduced neuronal viability. These data suggest that chronic high levels of homocysteine are not only a risk factor for migraine with aura but also may increase the risk of migraine stroke.

CSD is a transient wave of depolarization of neurons and glia followed by a significant shift in transmembrane ionic concentrations and cellular swelling. CSD is evolutionarily conserved in the central nervous system of a wide variety of species, from locusts to humans. Depolarization spreads slowly, at a rate of only a few millimeters per minute, through the gray matter, regardless of functional or vascular subdivisions, and lasts up to a minute in normal tissue. In a healthy brain, repeated CSD does not damage brain cells due to energy-dependent recovery of ionic gradients and cellular metabolism [34,35,36,37,38,39]. At the same time, CSD also occurs in various pathological conditions, including migraine with aura [40], cerebrovascular disorders [41], epilepsy [42,43], traumatic brain injury [44], and neurosurgical procedures [45]. The number of CSDs and the duration of the depolarization and repetition rates are closely related to the outcome of the patients [40,46,47,48]. On the other side, the possible neuroprotective effects of CSD were discussed due to its pre-conditioning effects when preliminary CSD generation reduced the stroke infarction size and cell death [49,50,51].

Increased plasma homocysteine levels have known neurotoxic effects and are considered a risk factor for coronary heart disease, peripheral vascular disease, stroke, age-related dementia, and neurodegeneration. Moreover, homocysteine directly activates glutamate receptors, including NMDA receptors, which are considered to be one of the main triggers of CSD occurrence and propagation [52,53,54,55,56,57,58,59].

In this study, we used a maternal or prenatal form of hHCY, in which females obtained a high-methionine diet before and during pregnancy, and further experimental work was carried out on their offspring that received a control diet. Along with oxidative stress, inflammation, mitochondrial dysfunctions, and a decreased level of expression and activity of CBS were shown in rats with prenatal hHCY [12]. CBS is the rate-limiting enzyme of the transsulfuration pathway utilizing homocysteine, and its deficiency is one of the common forms of hereditary homocystinuria in humans [60,61]. One of the frequently used models of hHCY is heterozygotes for CBS-deficient mice that have an approximately 50% reduction in CBS mRNA and enzyme activity in the liver and have twice the normal plasma homocysteine levels, corresponding to moderate hHCY [61]. In this study, rats with prenatal hHCY were exposed to high homocysteine levels throughout life, beginning from the perinatal period, and demonstrated moderate plasma homocysteine concentrations, which made it possible to study the effects of chronic hHCY in mechanisms of migraine.

In our previous studies, we provided evidence of higher sensitivity of rats with prenatal hHCY in the nitroglycerine-induced model of episodic and chronic migraine [18]. In addition, we observed higher excitability of meningeal afferents of the trigeminal nerve and isolated neurons from the trigeminal ganglion in rats with hHCY [62]. Finally, lower concentrations of KCl were necessary to induce CSD in the somatosensory cortex of rats with hHCY (for hHCY rats—0.06 M, for control rats—0.3 M KCl) [17]. However, the ability of the cortex of rats with hHCY to generate recurrent CSD, which was shown for different physiological and pathological conditions [63], was not investigated.

Our results demonstrated that the background neuronal spontaneous activity was higher in rats with hHCY, indicating higher excitability, similar to previous data [17,64]. Analysis of SEPs, which is widely used in clinical practice to evaluate the integrity of somatosensory pathways and functional testing [65,66], did not reveal differences between the control and hHCY groups, with the exception of latency to the peak of the SEP. The peak to the SEP reflects mainly the time to conduct sensory signals from the periphery to thalamic terminals in the cortex; therefore, the increase in time to peak indicates the increase in sensory processing time in rats with hHCY.

Epipial KCl application for 2 h was used to induce recurrent CSD. Similar to previous data in hHCY animals, the CSD was characterized by lower amplitude and longer rise time and half-width (T1/2) compared to the CSD in control animals [17]. At the same time, more CSDs during 2 h were generated in the hHCY group. Analysis of the vertical profile of CSD propagation showed that in control depolarization, waves spread across all cortical layers or were limited by supragranular layers. In rats with hHCY, subsequent CSD gradually shifted to the deeper layers of the somatosensory cortex, whereas superficial layers passed into a state of severe depression of activity. Those changes in the CSD propagation in the hHCY group may be associated with altered neuronal properties and reduced resistance to KCl-evoked depolarization.

Analysis of SEPs and MUA during recurrent CSD supported this proposal. KCl application on the surface of dura mater, along with generation of CSD, suppressed SEPs and MUA, which indicates inhibition of cortical activity. The recovery of SEPs and MUA was observed after the first CSD in the control group and only partially in the hHCY group. After the last CSD in the control group, SEPs and MUA were recovered in 30 min, and no recovery was observed in the hHCY group.

CSD has been shown to cause marked metabolic changes in brain tissue, such as a drop in tissue oxygen tension, formation of reactive oxygen species (ROS), and energy depletion, and depress neuronal activity [21,43]

The recovery of cortical activity after recurrent CSD depends on the restoration of ionic and energy homeostasis and the re-establishment of membrane potential. ATP consumption increases due to the activation of Na^+^-K^+^-ATPase and other ATP-dependent processes necessary to restore ion gradients across neuronal and glial membranes and to recycle and replenish neurotransmitters after their release during depolarization [34]. In energy-compromised tissue, spreading depolarization facilitates neuronal death, whereas, in healthy tissue, it is relatively innocuous [67].

Metabolic dysfunctions in hHCY include oxidative stress due to the production of ROS and a decrease in the activity of antioxidant enzymes (superoxide dismutase and glutathione peroxidase) in the brain [68,69,70,71]. Stimulation of neurons with homocysteine over a longer period leads to necrotic cell death [72,73,74]. The decrease in Na^+^/K^+^-ATPase activity was shown in the brain tissues of animals with hHCY [75,76].

Additionally, the activity of LDH in brain tissues of rats with hHCY was increased, which can be a consequence of homocysteine-induced ATP depletion and increased glycolysis [77,78]. ATP depletion was also associated with apoptotic cell death in glial cells and neuronal subpopulations [79], which can increase the serum level of LDH. As a result, the resistance of neurons of rats with hHCY to recurrent depolarizations and the ability to recover reduces, which was observed at the site of recording, especially in the supragranular layers. Moreover, TTC staining of brain slices after 2 h of recurrent CSD revealed a “necrotic funnel” in hHCY animals, reflecting the death of brain tissue. TTC staining is widely used to study the viability of cells in penumbral and infarction imaging [80].

In clinical conditions, a migraine attack occurring as migraine with aura proceeding for more than 60 min may induce migrainous infarction, a rare pathogenic event associated with an ischemic brain lesion demonstrated by neuroimaging [81,82]. The pathogenesis of migrainous infarction is not known; however, CSD, which increases energy consumption, was proposed as a key factor. Other factors contributing to migrainous infarction may include vascular, inflammatory, and endothelial dysfunctions, patent foramen ovale, gender, oral contraceptive pill use, and smoking [83]. hHCY is also a risk factor for stroke, including intracerebral hemorrhage [84], and increased homocysteine levels have been proposed to be used as a preclinical biomarker for stroke [85]. Even mild hHCY may increase the risk of clinical manifestations of stroke, due to the multiple biochemical effects of homocysteine, including endothelial dysfunctions, thrombosis, oxidative stress, and inflammation [69,86,87,88,89,90,91]. Therefore, chronic high homocysteine levels and recurrent CSD increase the risk of ischemic brain lesions.

## 5. Conclusions

According to our data, rats with moderate hHCY demonstrated higher sensitivity to recurrent CSD, higher cortex excitability, and more significant suppression of spontaneous and sensory evoked activity after CSD compared to the control. Moreover, no recovery of neuronal activity was observed in animals with hHCY. An impaired propagation of CSD in upper cortical layers, along with ischemic signs, according to TTC staining, makes it possible to suggest that recurrent CSD, together with elevated homocysteine levels, increases the risk of cortical damage. Therefore, our results proposed increased chronic homocysteine levels as a comorbidity for migrainous infarction in patients with migraine with aura, and the control of homocysteine concentrations in blood plasma can prevent migraine complications in clinical conditions.

## Figures and Tables

**Table 1:** Depth profile for MUA during and after sensory evoked potentials in the rat cortex.

Layers of the Cerebral Cortex	Control Group	hHCY Group	Control Group	hHCY Group
	MUA During SEP, spikes/s		MUA After SEP, spikes/s	
L2/3	2.02 ± 0.89	5.72 ± 2.11	6.11 ± 2.33	22.96 ± 9.39
L4	8.05 ± 1.55	8.04 ± 2.04	50.02 ± 18.46	49.16 ± 16.92
L5/6	8.27 ± 1.40	7.02 ± 1.28	63.03 ± 15.72	35.44 ± 9.02

## Data Availability

The raw data supporting the conclusions of this article will be made available by the authors on request.

## Abbreviations

hHCY	hyperhomocysteinemia
NMDA	N-methyl-D-aspartate
CSD	cortical spreading depression
H_2_S	hydrogen sulfide
KCl	potassium chloride
MMPs	matrix metalloproteases
TTC	2,3,5-triphenyltetrazolium chloride
DC	direct current
MUA	multiple unit activity
LFP	local field potential
SEP	sensory evoked potential
ATP	adenosine triphosphate
LDH	lactate dehydrogenase
ROS	reactive oxygen species
SOD	superoxide dismutase
GPx	glutathione peroxidase
